# The Relationship between Online and Offline Information-Seeking Behaviors for Healthy Nutrition

**DOI:** 10.3390/ijerph181910241

**Published:** 2021-09-29

**Authors:** András Fehér, Miklós Véha, Henrietta Mónika Boros, Bence Kovács, Enikő Kontor, Zoltán Szakály

**Affiliations:** 1 Institute of Marketing and Commerce, Faculty of Economics and Business, University of Debrecen, 4032 Debrecen, Hungary; borosh19@gmail.com (H.M.B.); kovacs.bence@econ.unideb.hu (B.K.); kontor.eniko@econ.unideb.hu (E.K.); szakaly.zoltan@econ.unideb.hu (Z.S.); 2 Naturtrade Hungary Ltd., 6725 Szeged, Hungary; vehamiki@gmail.com

**Keywords:** health, nutrition, online and offline information sources, attitude, eating habits, Hungary, university students

## Abstract

In addition to preserving their health, young people can also play a role in providing information to wider society. Nutrition habits that have developed over the years at college have an impact on the foundation of a lifelong lifestyle. Our aim was to identify university students’ online and offline information-seeking attitudes related to healthy nutrition and create a new theoretical concept. Participants were university students (*n* = 612), and the self-administered, paper-based questionnaires were sent out to nine Hungarian universities. Both descriptive and multivariate statistical procedures were used in the analysis. Online and offline information sources were categorized. In relation to university students’ information-seeking competence, the component of electronic health literacy was determined. In analyzing attitudes, the components of acceptance of, incentive for, and rejection of or ambivalence towards healthy nutrition were identified. The information-seeking categories related to the stages of university students’ conscious transition to healthy nutrition were also identified. University students’ competences related to electronic health literacy are essentially favorable. This target group accepts healthy nutrition and tries to recommend it to others, too. However, a rejecting or ambivalent attitude could also be identified. Online and offline sources of information accompany university students’ transition of the relevant stages of changes. The theoretical concept that we developed can contribute to bridging gaps in the interrelatedness of diverse information sources and healthy nutrition.

## 1. Introduction

The WHO [1] defines health as a state of complete physical, mental and social well-being and not merely the absence of disease or infirmity. Felman [2] claims that the balance of mental health (in addition to a lack of depression and anxiety, the ability to enjoy life and a feeling of safety) and physical health (regular physical activity and a balanced diet) is crucial to the reduction in the risk of various diseases. It is beyond dispute that health is one of the current megatrends. In their spending, the individuals’ primary aims are no longer to cure diseases, but to prevent them with some products or services [3,4,5,6]. In most cases health can be identified with a healthy lifestyle. This statement is confirmed by the research of Holly et al. [7] in which it is suggested that a healthy lifestyle is one of the most determinant factors in an individual’s intention to improve health. Von Bothmer and Fridlund [8] demonstrated that the health behavior of Swedish university students was most determined by the nature of their healthy lifestyle. It is important to state that 16.3% of Hungarians perceived their lifestyles as very good, while in the countries of the EU 21.3% of the population had a similar opinion [9]. Conscious transition to and maintenance of a healthy lifestyle depend on a number of factors, with one of the most important being the development of an appropriate diet [10,11,12,13], and lack of information about a diet which is considered healthy emerges as a typical confounding factor [14]. During the transition to healthy living, recommendations by friends, the opinions of nutrition professionals and peer pressure among young people can have positive effects [15,16,17]. Taking global tendencies into consideration, in 2016, over 1.9 billion people were overweight (18 years of age and older), of whom over 650 million were obese. In the group of children and adolescents between 5 and 19 there were over 340 million who were in the overweight or obese category [18]. Among non-communicable diseases diverse chronic diseases (e.g., cardiovascular diseases and tumors) can be identified as one of the major problems of 21st century society. One of the root causes of these diseases is inappropriate diet [19]. Hungary is in the leading group for mortality trends from cardiovascular diseases since, as per 2016 WHO data, close to half of all deaths (47%) were caused by these diseases [20]. The various chronic diseases primarily threaten older people [21]. The role of younger age groups is of major importance in the fight against the above diseases, as they are able to prevent more severe problems with the help of a more conscious and healthy lifestyle [22,23,24]. 

The relationship between health and nutrition has been a well-known scientific and public issue for decades now [25]. Healthy eating means the regular consumption of a variety of foods and drinks, which, if consumed in appropriate ways and ratios, can contribute to the reduction in the risks of certain diseases. A healthy diet provides the body with adequate nutrition, vitamins, and minerals. Creating a varied diet is crucial in preventing health problems caused by states of deficiency and excessive intake. Thus, consumption of foods considered healthy, including vegetables and fruit, and increasing the consumption of fish, wholemeal cereals (e.g., buckwheat, millet, and quinoa) is especially recommended, as is increasing the consumption of low-fat milk and dairy products. Furthermore, the consumption of animal fats and foods with high sugar and salt content should be reduced [26,27,28,29,30].

In many cases, healthy eating is not a priority among university students yet, as eating is a source of pleasure, and tastes, commercials, advertisements and fads considerably influence their food-buying and consumption preferences. For them, food appears as a status symbol and less as a source of nutrition [22,31]. Other researchers have addressed the knowledge of university students about nutrition in several aspects (predicting variables and phenomena). In analyzing first-year students in Kuwait, El-Sabban and Badr [32] found that threshold of adolescence and young adulthood, young people’s attitude to food is affected by several environmental (the knowledge of nutrients) and cultural (practical or theoretical type of colleges) factors. In their research on Thai students, Tanvatanagul and Uaphanthaseth [33] concluded that their main concern in switching to a healthier lifestyle was motivated by their desire to improve their bad eating behavior. Further research was conducted into the omission of the main meals (most frequently breakfast) among university students, along with low consumption of vegetables, fruit and dairy products [34,35,36,37]. Tavolacci et al. [38] examined French university students’ eating disorders. It has been found that students with eating disorders are more likely to seek the advice of their doctor for symptoms of stress and anxiety than students without eating disorders. Gergely et al. [39] drew a parallel between the health-conscious attitude of Hungarian university students and regular exercise, conscious shopping, and a life free from harmful compulsive activities. Their findings are important because, among other things, the university years are a particularly critical period in terms of laying the foundation for a lifelong lifestyle. A body of research shows that the lifestyle developed during university years considerably determines the emergence or avoidance of chronic diseases [40,41,42,43].

It has been established that certain factors influence an individual’s transition and adherence to healthy eating. Walter and Skerrett [44] refer to a lack of financial resources as a factor preventing the switch to health-conscious eating. Gál et al. [45] argue that consumer’s associations (e.g., diet, slimming cure) related to healthy eating significantly affect actual behavior. Based on this, it can be concluded that credible information is of the utmost importance because the majority of consumers have insufficient or poor basic knowledge in the field of healthy lifestyle [46]. All the information available about healthy eating makes it difficult for consumers to identify accurate information. Providing consumers with knowledge is a crucial factor, which, if complemented with motivation, can contribute to changing eating-related attitudes, which, in turn, can lead to more conscious eating [47,48].

Prior information-seeking, which enables the individual with a preference for a potential for lifestyle transition to make informed decisions, is a prerequisite for health consciousness. This can be compared to the definition of health literacy. Health literacy: “it is linked to literacy and entails people’s knowledge, motivation and competences to access, understand, appraise, and apply health information in order to make judgments and take decisions in everyday life concerning healthcare, disease prevention and health promotion to maintain or improve quality of life during the life course.” [49] (p. 3). The concept of electronic health literacy can be identified as the appearance of the above factors on various internet platforms, like health websites [46]. In order to improve the low levels of health knowledge, the integration of gamification into various health applications (e.g., fitness tracking, nutrition diaries) can be beneficial. This method increases health knowledge or understanding the full functionality of apps [50]. The population of Hungary can be classified into the following levels of health literacy: insufficient (20%); problematic (32%); sufficient (38%); and excellent (11%). Based on the aggregate index (32.3%) Hungarians occupy the middle ground among the European countries examined [51].

The traditional sources of information (e.g., television, radio, the press, and personal consultation with a specialist) are being increasingly complemented and replaced by new and novel digital opportunities to obtain information. In this way consumers can form relevant and often customized approaches when deciding what product they should choose which would be the most beneficial to them. This relationship is particularly true in the case of shopping for food. There are indications that digitalization significantly affects the shaping of the attitudes of consumers toward food consumption. This is most evident in the online search for food information (e.g., scanning QR codes on a product package with a mobile phone or using search sites) [52]. In Japan, a separate campaign has been launched in which Society 5.0 is used to recommend different health and lifestyle values to individuals [53]. The increase in the importance of digitalization can be compared to people’s Internet use habits. On a global level, 64.7% of the population can be considered active Internet users, while the corresponding ratio in Hungary is 90%, (similar to EU data) [54]. A considerable proportion of our time on the Internet is spent searching for various types of content. Eurostat [55] data show that 63% of Internet users in Hungary search for health-related content (diseases, injuries, eating and health improvement). The corresponding ratio in the populations of EU27 countries is 55%.

Digital health shows parallels with technologies that have an effect on the provision of information and the improvement of health literacy. A new phenomenon, we call it “digital health” and define it as “the cultural transformation of how disruptive technologies that provide digital and objective data accessible to both caregivers and patients leads to an equal level doctor-patient relationship with shared decision-making and the democratization of care” Meskó et al. [56] (p. 1) [57,58]. The efficiency of mobile applications in nutrition-related decisions is ever more important. Due to digitalization, new methods of nutritional assessment are developing fast [59].

Seung-Dae and Myung-Gwan [60] investigated South-Korean university students’ search for health-related information. They found that information-seeking on the Internet is much more efficient than use of the traditional channels of information on account of its real-time nature. Achampong et al. [61] examined university students’ search for health-related information on social media platforms. They concluded that students find it easy to gain access to health-related information and most of them are happy to rely on it when making decisions about their health. 

The main objective of this research is to use a theoretical concept with empirical results to identify university students’ online and offline information-seeking behavior for healthy nutrition. In our analysis, we created categories based on the perceived significance of online and offline sources of information. Then we compared these with electronic (digital) health literacy related to healthy nutrition as well as various attitudes and competences. We included variables of subjective perception in relation to healthy nutrition in the relationship. Based on preliminary research, we concluded that there is a relative scarcity of research into university students’ healthy nutrition internationally, and particularly in Hungary. The majority of research investigates generations younger than university students. Another reason why we decided to investigate university students’ attitudes is that, in using sources of information, online channels of communication (particularly social media platforms) appear to be increasingly important compared with traditional or offline channels [61,62]. Our hypothesis is that, in searching for information related to healthy nutrition, university students do not only lay the foundation for a transition in their own behavior but can even act as intermediaries, imparting information from themselves to other generations.

## 2. Materials and Methods

### 2.1. Sampling Procedure and Method of Data Recording

In our research, a quantitative questionnaire survey was performed between January and March 2020. The Faculty Ethics Committee of the Faculty of Economics of the University of Debrecen examined and approved the research from an ethical point of view (reference number: GTKDH/71/2021). To elicit answers, a paper-based self-administered questionnaire was used. After prior consultations, the questionnaires were sent to the 10 university campuses around the country where students of faculties with an economic sciences profile were targeted. Preliminary consultations mean that we had formally contacted the leaders of the selected university faculties and asked permission to conduct research among their students. We also asked for their assistance in distributing the questionnaires. The ten largest universities by student population in Hungary were included in the research. The reason for this is to maximize our selection opportunities from the undergraduate target group. The reason for choosing the economics-oriented courses is that our research group also represents this field of science, thus we considered the research in this field has been considered to be the most relevant for us. Our target group consisted of students from bachelor’s, master’s and university level vocational training. One hundred questionnaires were sent to each university campus. Roughly the same number of questionnaires were returned from nine campuses. A total of 1000 paper-based questionnaires were sent out during the questionnaire research. Of these, 630 were returned and 612 were appropriate for evaluation. At the time of the study, the COVID-19 pandemic situation was already underway and, because of the epidemiological measures, large numbers of questionnaires could not be completed. To preserve the original concept of the research, we did not switch to conducting the survey online for the rest of the questionnaires. Among the topics examined in the questionnaire, the subjective perception of students’ healthy eating, attitudes, and channels of information used in information-seeking for healthy nutrition, as well as the types of their use, were analyzed.

### 2.2. Sociodemographic Background of the Sample

Table 1 summarizes the sociodemographic features of the university students surveyed. In the sample we see a higher number of females and those living in municipalities. The age distribution of the students can be regarded as balanced, but 20 and 21-year-olds were in the majority. The majority of the respondents were bachelor-program students at the University of Debrecen. The majority of respondents spend an average of 3 to 5 h a day using the Internet. In terms of their subjective income perception, the majority of the students and their families live well on the income at their disposal and can even save some of it. The respondents regard themselves as being moderate-level health-conscious. The BMI index of the overwhelming majority of the students is in the normal range. Outside of the gender variable, concerning the other sociodemographic features, no significant differences were found as a function of the elements of the theoretical concept.

### 2.3. Adapted Models

The following section gives a detailed description of the types of earlier models and research segments used or adapted during the research to create our own theoretical concept.

#### 2.3.1. Importance of Online and Offline Sources of Information Related to Healthy Nutrition

The sources of information were examined by the categories differentiated by Szűcs et al. [48] based on their importance, complemented with traditional and Internet-based sources. Respondents could evaluate the importance of information on a scale of 1–5 (1—not important at all; 5—extremely important). In the questionnaire, the importance of 20 different sources of information was examined in relation to information-seeking for healthy nutrition. In terms of the nature of the sources of information, we identified online (6 elements) and offline (14 elements) categories. Section 3.1 provides a detailed discussion of the relevant elements of the two categories. In creating the theoretical concept, data reduction of the measurement variables for the examination of online and offline categories was performed by calculating the arithmetic mean, which has the advantage of reducing the weight of relative errors in the individual responses [63].

#### 2.3.2. Subjective Perception of Healthy Nutrition—Time Interval of Online and Offline Information-Seeking

The subjective perception of healthy nutrition was incorporated into the concept through two variables. The perception of the aggregate time interval of information-seeking for healthy nutrition spent on online and offline forums was included in our research, based on works by Jepsen [64], (Internet Search Model, ISM) and Fehér et al. [52]. Information-seeking was examined on a 5-point ordinal scale with the following statements: “A very long time spent on online searching”; “A relatively long time spent on online searching”; “A similar length of time spent on offline and online searching”; “Relatively long time spent on offline searching”; “A very long time spent on offline searching”. During the analysis, frequency distribution was calculated.

#### 2.3.3. Relative Perception of Healthy Nutrition—Transtheoretical Model (TTM) of Change in the Case of Transition to Health-Conscious Nutrition

**Figure 1:**
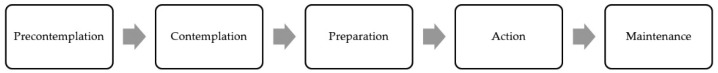
Stages of the transtheoretical model of change.

The transition to health-conscious nutrition was examined using the transtheoretical model (TTM) of change on a 6-point ordinal scale [65,66,67]. Using the TTM model, questions were asked about the different stages that the university students involved in the research represented in their transition to healthy nutrition. The analysis was performed using the five stages of the TTM model (Figure 1). The statements about the relevant stages in the transition to healthy nutrition were determined based on research by Szabó [67]. The interpretation of the data needs to be complemented with the proviso that what we have in question here in practice is nutrition deemed healthy by the consumer. This is independent of how healthy nutritional science considers a particular way of eating.

#### 2.3.4. Components of Attitudes towards Healthy Nutrition and of Electronic Health Literacy

Internet searches for healthy nutrition were analyzed using the electronic (digital) health literacy scale (eHealth Literacy Scale, e-HLS) identified by Britt et al. [68]. The original and extended versions of the scale are used extensively by researchers [69,70,71,72]. The 8 statements used in the analysis were translated into Hungarian and were complemented with expressions related to healthy nutrition. The statements are described in Section 3.4. Respondents could rate the factors on a scale of 1–5 (1—not characteristic at all; 5—entirely characteristic). 

In analyzing attitudes towards healthy nutrition, respondents had to rate the 23 statements compiled based on works by Szűcs et al. [48], Szabó et al. [73], and Szakály et al. [12]. The university students responded to the statements on a scale of 1–5 (1—do not agree at all; 5—strongly agree). The statements were as follows: 1. It is impossible to provide a common recipe for healthy nutrition for everyone, since everyone needs to pay attention to different things; 2. We know far more about what we should change to eat healthily, but, unfortunately, we do little to achieve it; 3. For a healthy person it is sufficient to pay attention to having variety, balance and moderation in nutrition; 4. However healthy something is said to be, I don’t force it if I don’t like it; 5. Healthy eating is not about punishing ourselves, on the contrary, it is a source of pleasure; 6. Phytosterols, flavonoids, antioxidants, probiotics—one is at a loss for choice, there are so many unknown concepts and one is becoming more and more puzzled as to what to do if one intends to eat healthily; 7. Healthy nutrition is very costly; 8. Knowledge about healthy nutrition keeps changing and expanding, thus it is difficult to keep up with it; 9. What tastes good makes me really healthy; 10. I have tried several times to eat healthily but, after temporary self-punishment, I have returned to my usual ways; 11. Healthy nutrition always reminds me of slimming cures; 12. I eat a perfectly normal diet so my body is automatically provided with whatever I need; 13. I only eat what I like, even if it is considered less healthy; 14. I try to create a balanced diet; 15. I make a conscious effort to avoid foodstuffs with ingredients that I consider harmful; 16. I find it important that the people around me should also have a healthier diet; 17. I make a conscious effort to find foodstuffs with ingredients that I consider beneficial; 18. I pay attention to the effect of foods and foodstuffs on body weight; 19. I make a conscious effort to avoid foods that are energy-dense (oil, sugar); 20. I pay close attention to healthy and balanced nutrition and choose my foods carefully; 21. I do not consider unhealthy nutrition a problem; 22. I persuade others, too, to eat a healthier diet; 23. When compiling my diet, I follow nutrition-related recommendations based on current medical knowledge.

### 2.4. Preliminary Theoretical Concept of the Relationship between Online and Offline Information-Seeking Attitudes for Healthy Nutrition

**Figure 2:**
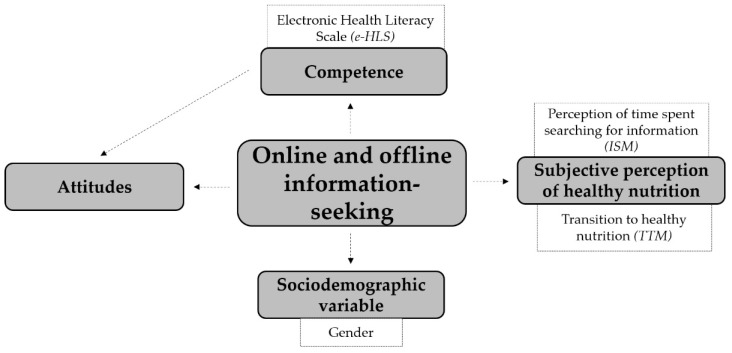
Preliminary interrelatedness of the theoretical concept.

During the creation of the theoretical concept (Figure 2) the connections between university students’ online and offline information-seeking related to healthy nutrition were analyzed in relation to the subjective perception of healthy nutrition, and the competence of health literacy and attitudes towards healthy nutrition. As a control variable, the sociodemographic variable, distribution by gender, was included. The relationships among the components of the theoretical concept were analyzed using an examination of statistical relationships.

The online and offline categories of information sources were analyzed with the help of components, while the relationships among the components were examined using Pearson’s linear correlation. A moderate negative correlation can be identified if the values are −0.7 < r < −0.2. Values of 0 < r < 0.2 indicate a weak negative correlation while values of 0.2 ≤ r < 0.7 indicate a moderate positive relationship [63]. The demographic variable of gender was examined using the Mann–Whitney’s U test, and the questions analyzing the subjective perception of healthy nutrition were compared with the aggregate online and offline categories of sources of information using the Kruskal-Wallis test. Significant differences were found between the factors in each examined relationship.

### 2.5. Data Analysis

Evaluation of the questionnaires was conducted using IBM SPSS 25.0 statistical software (IBM, Armonk, NY, USA). For the analysis, frequency analysis, the chi square test, variance analysis (ANOVA), Mann–Whitney’s U and Kruskal–Wallis tests, and Pearson’s correlation calculation were used [74,75], while for the individual relationships, factor analysis and principal component analysis were applied. Our theoretical concept has not been validated using confirmatory factor analysis (path model). Only simple correlation coefficients were provided.

## 3. Results

This section provides a detailed description of the elements of the preliminary theoretical concept outlined above (Figure 2).

### 3.1. Significance of Online and Offline Sources of Information

The next section lists students’ categories of sources of information related to healthy nutrition, together with the mean values of their significance. The online group comprises six items (Table 2): website entries by dietitians and health sciences specialists (mean value = 3.64); search engines (3.10); blogs and forums (3.02); Internet news sites (2.99); social networks (2.86); online video channels by influencers (2.46). The offline category included 14 items (Table 3): personal information received during health care (physician, dietitian, pharmacist, etc.) (4.49); books and journals (3.99); food marking and label information (3.96); information provided by skilled shop assistants in specialized shops (e.g., organic shop, phyto-shop) (3.96); recommendation by acquaintance or friend (3.61); health-education publications (3.60); knowledge learned at school (3.43); magazines (3.20); product information and leaflets (2.83); free publications (2.83); television programs (2.48); television commercials (2.32); posters and printed advertisements (2.31), as well as radio programs (2.30). For university students it was a competent specialist (physician, dietitian, or pharmacist) who represented the most important source in terms of both online and offline sources of information.

### 3.2. Time Interval of Online and Offline Information-Seeking

**Figure 3:**
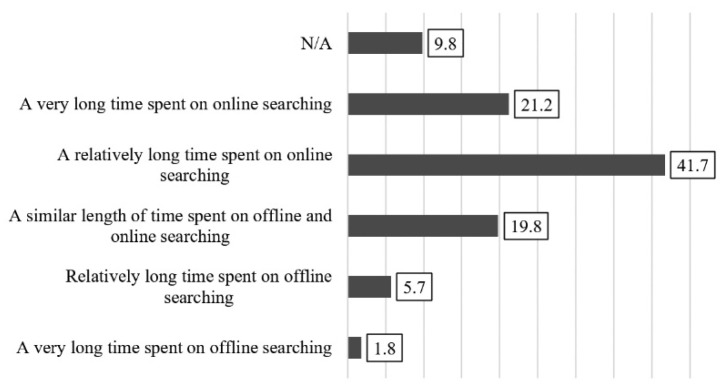
Perception of time spent searching for information related to healthy nutrition on online and offline platforms, % (*n* = 612).

Figure 3 shows the perception of the time spent on online and offline platforms during information-seeking for healthy nutrition. Some 41.7% of the university students surveyed spend somewhat more and a further 21.2% spend considerably more time doing online searching. A total of 19.8% of respondents think that they spend equal amounts of time seeking information on- and offline. Only 7.5% of university students search for information browsing traditional sources, with 5.7% of them spending slightly more, and 1.8% spending far more time doing this activity. In addition, 9.8% of the students surveyed did not respond or were unable to assess the question.

### 3.3. Transtheoretical Model (TTM) of Change for Transition to Health-Conscious Nutrition

**Figure 4:**
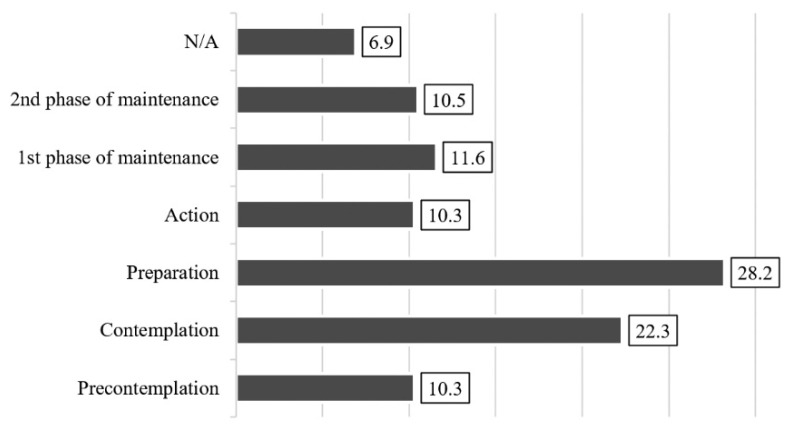
Perception of the individual’s transition to healthier nutrition, % (*n* = 612).

We used the transtheoretical model (TTM) of change to analyze the stages of the transition by university students to nutrition considered healthier (Figure 4). A total of 10.3% of university students are totally passive and can be classified as belonging to the “pre-contemplation” stage (“I do not intend to switch to nutrition I consider healthier in the next six months”). Of the students, 22.3% are at the stage of “contemplation”, which means that the possibility of transition emerges in their thinking (“I feel a strong urge to switch to nutrition that I consider healthier”). Then, 28.2% of the respondents thought that they were going to take concrete steps, placing them in the “preparation” stage (“I am going to take steps to switch to nutrition that I consider healthier within the next month”). In the meantime, 10.3% of them entered the “action” stage, after taking concrete steps recently (“I have followed a healthier type of nutrition for at least six months”). In addition, 11.6% of students at the first stage of “maintenance” have been eating healthily for some time (“I have been following healthy nutrition for more than six months now, and the chances of relapsing to my old eating habits are minimal”), and a further 1.5% at the “maintenance” stage “have always eaten healthily”. In all, 6.9% of the respondents did not answer the question. In sum, we can conclude that 32.4% of the university students can be classified as belonging to the “action” and “maintenance” stages.

### 3.4. Components of Attitudes towards Healthy Nutrition and of Electronic Health Literacy

In the first stage we investigated the structure of the two series of statements described in Section 2.3.4. using exploratory factor analysis (EFA). When examining electronic literacy, we retained all eight statements and identified one factor. In mapping attitudes, we performed multi-round data reduction. Scale reliability was examined using Cronbach’s alpha formula, and in the end, to optimize reliability, 4 variables were removed from the analysis. A further 5 factors were removed when creating factor structures due to low factor loading. After data reduction 3 factors were formed. Data reduction was carried out in the next step using separate principal component analyses (PSA) for every dimension, based on the previously determined structures in order to reduce variance from cross-loadings not explained by the indicators [63]. As a result, we obtained relevant variables for further correlation investigations. 

Table 4 contains the elements of the component electronic health literacy (C_EHL_) related to healthy nutrition (Cronbach Alpha = 0.868; KMO = 0.916; Chi-Square = 1776.2; df = 28; *p* < 0.001). It can be observed that greater component weight is attached to those factors that highlight the nature of information-seeking for healthy nutrition while proportionally lower component weights can be assigned to the differentiation and evaluation of the relevant information sources. The factors of the components all had relatively high component weights and the differences between the factors were minimal. This tendency is likely to confirm the fact that the competences of university students related to electronic health literacy are essentially favorable.

We identified three components in connection with university students’ attitudes to healthy nutrition; the relationships between these components are presented in Table 5. The first component is the acceptance of healthy nutrition (C_AHN_) (Cronbach Alpha = 0.841; KMO = 0.873; Chi-Square = 1476.1; df = 21; *p* < 0.001). The factors representing conscious knowledge of ingredients necessary for healthy nutrition have greater component weight than any effort to avoid some harmful ingredient. Among the variables, the following nutrition recommendations received the lowest component weight. The second component was the incentive to have healthy nutrition (C_IHN_) (Cronbach Alpha = 0.705; KMO = 0.500; Chi-Square = 213.8; df = 1; *p* < 0.001). The two factors in the component, with similarly high component weight, express an incentive to perform the transition to healthy nutrition in relation to the individual’s social environment. The third component is rejection of healthy nutrition or an ambivalent attitude towards it (C_RHNAA_) (Cronbach Alpha = 0.610; KMO = 0.650; Chi-Square = 335.0; df = 10; *p* < 0.001). The factors classified as belonging to the components have a dual interpretation. The relevant factors share a common feature: they have a detrimental effect on the transition to healthy nutrition. The component is significantly (*p* < 0.001) more typical of females than of males.

### 3.5. Theoretical Concept of the Relationship between Online and Offline Information-Seeking Attitudes Related to Healthy Nutrition

**Figure 5:**
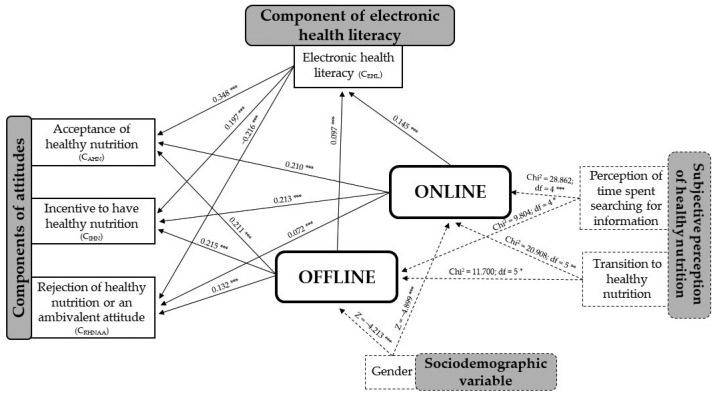
Theoretical concept of the relationship between online and offline information-seeking attitudes related to healthy nutrition. Correlation coefficient (r) values. Significance: * *p* ≤ 0.05; ** *p* < 0.01; *** *p* < 0.001.

The statistical relationships between online and offline information-seeking attitudes related to healthy nutrition are outlined in the theoretical concept in Figure 5.

There is a moderately strong positive linear correlation between the two components C_AHN_ and C_EHL_ (r = 0.348; *p* < 0.001). This leads us to conclude that electronic health literacy has a positive effect on the acceptance of health-conscious nutrition. There is a weak positive linear correlation between the components C_IHN_ and C_EHL_ (r = 0.197; *p* < 0.001). This means that, although a relationship can be detected between electronic health literacy and the incentive for healthy nutrition, it is relatively low. There is a moderately strong negative linear correlation between the components C_RHNAA_ and C_EHL_ (r = −0.216; *p* < 0.001). This leads us to conclude that the attitude of rejecting or being ambivalent about healthy nutrition shows a contradictory relationship to the component of electronic literacy.

Component C_EHL_ shows a weak positive correlation with both online (r = 0.145; *p* < 0.001) and offline (r = 0.097; *p* < 0.001) information-seeking categories. In all probability, this somewhat surprising finding came about because the competence of electronic literacy is essentially favorable for university students, hence the significance of online and offline possibilities of information-seeking does not function as a differentiating factor in this age group.

There is a medium strong linear correlation between the significance of the C_AHN_ component and of online (r = 0.210; *p* < 0.001) as well as offline (r = 0.211; *p* < 0.001) information. We identified a correlation similar to the previous one as a result of comparing online (r = 0.213; *p* < 0.001) and offline (r = 0.215; *p* < 0.001) information-seeking factors on the one hand, and C_IHN_ on the other. We can conclude that the attitudes of accepting healthy nutrition and the incentives thereof appear in a similar way in relation to the significance of various information sources, thus they are less easy to differentiate. The perceived significance of component C_RHNAA_ and online (r = 0.072; *p* < 0.001) as well as offline (r = 0.132; *p* < 0.001) groups of information sources show a weak positive linear correlation.

We used the Mann–Whitney’s U test to determine the significance of online (Z = −4.899; *p* < 0.001) and offline (Z = −4.213; *p* < 0.001) categories of information sources in relation to the control variable of gender. The higher ranking average of female university students reveals the fact that categories of online (ranking average = 334.46) and offline (330.58) information sources are more important to them than to male students (online: 262.86 and offline: 268.92, respectively). In other words, when searching for information on healthy nutrition, women attach more importance to relevant sources than men do, irrespective of their type. 

We used Kruskal–Wallis test to analyze the significance of the relationship between the categories of online (Chi^2^ = 28.862; df = 4; *p* < 0.001) and offline (Chi^2^ = 9.804; df = 4; *p* < 005) information sources in relation to the aggregate time spent searching for information on healthy nutrition on online and offline platforms. Online information sources appear to be more important to university students who spend slightly more (ranking average = 272.32) or considerably more time (332.93) searching for information on the Internet. On the other hand, offline sources of information are important to university students spending slightly more time (334.39) searching for information in a traditional way.

We used the Kruskal-Wallis test to determine the significance of the online (Chi^2^ = 20.908; df = 5; *p* < 0.01) and offline (Chi^2^ = 11.700; df = 5; *p* < 0.05) categories of information sources in relation to the relevant stages of the transition to healthy nutrition. The increasingly higher ranking averages lead us to conclude that, irrespective of the type of groups (online or offline) of information sources, these information sources play an increasingly large role in the relevant stages of university students’ transition to healthy nutrition (precontemplation, contemplation, preparation, action, and maintenance). Students consider offline sources of information to be more important in the “pre-contemplation” (ranking average = 242.43) and “contemplation” (292.65) stages; however, the highest ranking average in connection with the category can be identified at the “action” stage (318.45). At the two “maintenance” stages—“has had healthy nutrition for more than six months” (319.66) and “has always had healthy nutrition” (253.44)—online sources are more important. In the “preparation” stage online (299.75) and offline (295.15) categories of information sources are equally important. The results suggest that the relevant stages of the transition to health-conscious eating are influenced by different information sources. In the early stages the importance of offline sources is relevant whereas the later stages are characterized by the importance of online sources of information.

## 4. Discussion and Conclusions

During our research we identified the relationships between university students’ online and offline information-seeking attitudes for healthy nutrition using theoretical concepts with empirical contexts. We determined each element of the concept based on previous research, adapting it in certain cases to the objectives of our own research. Our main aim was to create a new theoretical concept; thus, we did not think it reasonable to compare our findings with the findings of the original research in terms of every element of the theoretical concept. One of the reasons why we decided against comparing our new results with earlier results was that the target groups in previous research were different from our target group of university students. Our concept was based on a national large-sample questionnaire survey. The research was exploratory in nature, and our survey is not representative. Nevertheless, we believe that the relationships in the theoretical concept have provided us with valuable data in relation to the Hungarian student population. In the survey, students of nine Hungarian universities were targeted with paper-based questionnaires, of which a total of 612 could be evaluated.

Relying on their own subjective perception, 62.9% of university students tend to get their information from online sources [52,64]. According to the transtheoretical model of change (TTM) [65,66,67], one third (32.4%) of university students are at the “action” and “maintenance” stages in their transition to healthy eating. All other university students (50.5%) are at the “contemplation” or “preparation” stages, which means that they intend to change their eating habits. 

We determined online (6 elements) and offline (14 elements) information sources [46], which were used as a basis for studying the interrelationships of the concept. 

With regard to university students’ information-seeking perceived skills we determined the electric health literacy (C_EHL_) component. It is generally true that the target group’s perception of skills related to electronic health literacy is essentially favorable. We identified three components when identifying university students’ attitudes toward healthy nutrition. Acceptance of healthy nutrition (C_AHN_) and incentive for healthy nutrition (C_IHN_) suggest favorable attitude features. The C_AHN_ component proves that healthy nutrition is essentially important for the majority of university students. The C_IHN_ component confirms Szakály’s [17] earlier statement that young people’s role as advocates in a community is important when it comes to imparting the values of the transition to a healthy lifestyle. The component of rejection of healthy nutrition or an ambivalent attitude (C_RHNAA_) incorporates the confounding factors that emerge in students’ healthy eating.

The possibilities of online and offline information-seeking show similar importance in the components of accepting healthy nutrition (C_AHN_) and of incentives for healthy nutrition (C_IHN_). However, in the case of rejection of healthy nutrition or an ambivalent attitude (C_RHNAA_) component, the sources of information are less decisive, a tendency that assumes that these attitudes may be more deeply rooted in rejectors, they may derive from childhood or may be of family origin. It can be concluded, then, that the perceived significance of online and offline categories of information sources is clearly related to the type of the platforms used in information-seeking in relation to their time periods.

The role of online and offline sources of information is more prominent in female university students. The result is not surprising, as the role of women in later stages of the life cycle (e.g., within the family) is also crucial in developing a healthy diet. It can be concluded that there is a particular focus on the different ways of information-seeking in the process of the transition to healthy nutrition, irrespective of their type. Offline sources are mainly important at the initial stages (“precontemplation” and “contemplation”) while online sources tend to be more significant at the “maintenance” stage. Thus, in all likelihood, university students lay the foundation for the change in their behavior using offline sources, which they later strengthen with the help of online sources or complement their knowledge. This is also indicated by the result that the young people rated the “personal information received during health care” as the highest of the offline information sources but marked product labels and specialty store assistants as important sources. So, it seems that information gained through direct personal experience is later supplemented with online information sources. The importance of online information sources in relation to university students’ information-seeking for health has been highlighted by previous research as well [60,61,62].

We believe that the theoretical concept that we devised during our research can contribute to bridging gaps in the interrelatedness of various sources of information and healthy nutrition. There are limitations to the comparability of the findings of our research with those derived from other research since the composition of the concept can be regarded as unique. This theoretical concept is a conceptual variant and can thus be complemented by further research and this concept can be subjected to validation. The framework and important elements of this concept are outlined in this study. We made a complex analysis of the information-seeking related to healthy nutrition in the university student age-group. As a result of this analysis, our analyses revealed the interrelationships of our own approach. It is important to note that the theoretical concept can be relevant in measuring not only university students’ attitudes but those of other target groups, too. 

The limitations of our research are the non-representative (the Hungarian student population) nature of our sampling and our failure to reach the original sample size of 1000 as per our original plans. It is difficult to achieve representative sampling among the Hungarian university student population. The reason for this is that, in recent years, the institutional structure of Hungarian universities has undergone major changes and is still undergoing continuous changes today, too. As a consequence, we were unable to create relevant quotas based on the students’ sociodemographic profiles in the universities involved in the research. At the time of the survey the COVID-19 pandemic situation was already underway, and had a direct effect on the completion of the questionnaires, too. However, to retain the relevance of our research, we did not switch to an online survey instead of the paper-based one. Additional limitations of the research are that the survey resulted in self-reported data. One target group was interviewed, and there was no cross-sectional research. Our theoretical concept has not been validated using confirmatory factor analysis (path model). Only simple correlation coefficients were provided. 

## Figures and Tables

**Table 1:** Sociodemographic background of the sample.

Description	Distribution of the Sample	
	Number	%
By all students surveyed		
Total	612	100.0
By gender		
Female	372	60.8
Male	240	39.2
By type of residence		
Capital	50	8.2
Municipality	228	37.3
Other towns	182	29.7
Village	152	24.8
Age distribution		
18–19	153	25.1
20–21	269	44.0
Over 22	189	31.0
Distribution by level of education		
Bachelor program	332	54.2
Master’s program	59	9.6
University vocational program	221	36.1
Distribution by qualifications		
University of Debrecen	115	18.8
University of Debrecen– Szolnok Campus	81	13.2
University of Kaposvár	42	6.9
Széchenyi István University	94	15.4
Budapest Business School	98	16.0
Szent István University	14	2.3
University of Nyírgyháza	95	15.5
University of Pécs	9	1.5
Perception of time spent using the Internet		
Less than 30 min	3	0.5
Between 30 min and 1 h	27	4.4
Between 1 and 2 h	121	19.8
Between 3 and 5 h	344	56.2
Between 6 and 10 h	93	15.2
Over 10 h	12	2.0
Perception of subjective income position		
Live very well off the income and can even save	182	29.7
Can live off the income but can save only a little	290	47.4
The income is barely sufficient to live on and the family cannot save any of it	85	13.9
Sometimes the income is not sufficient for the family	7	1.1
Family faces regular daily financial issues	4	0.7
Does not know / Does not answer	44	7.2
Subjective health consciousness		
Not health conscious at all	11	1.8
Mostly not health conscious	76	12.4
Half health conscious, half not	252	41.2
Mostly health conscious	232	37.9
Very health conscious	35	5.7
By BMI index		
Thinness (<18,5)	54	9.1
Normal (18.5–25)	406	68.5
Overweight (25–30)	108	18.2
Obesity (>30)	25	4.2

**Table 2:** Examination of online sources of information related to healthy nutrition based on their importance (*n* = 612).

	Mean	Median	Mode	Deviation	Skewness
Homepage entries by dietitians/health science specialists related to healthy nutrition	3.64	4	4	1.096	−0.566
Search engines (e.g., Google, Bing)	3.10	3	3	1.052	−0.121
Blogs, forums	3.02	3	3	1.015	−0.086
Internet news sites (e.g., BBC, CNN)	2.99	3	3	1.067	−0.079
Social media (e.g., Facebook, Instagram)	2.86	3	3	1.151	−0.062
Online video channels by influencers (e.g., YouTube channels)	2.46	2	1	1.203	0.349

**Table 3:** Examination of offline sources of information related to healthy nutrition based on their importance (*n* = 612).

	Mean	Median	Mode	Deviation	Skewness
Personal information received during health care (physician, dietitian, pharmacist, etc.)	4.49	5	5	0.802	−1.710
Books, journals	3.99	4	5	1.105	−1.010
Food labelling/marking, label information	3.96	4	4	0.897	−0.609
Information provided by skilled shop assistants in specialized shops (e.g., organic shop, phyto-shop)	3.96	4	4	0.916	−0.786
Discussion with neighbor, acquaintance,	3.61	4	4	1.038	−0.585
Health booklets, health education handbooks	3.60	4	4	1.068	−0.491
Knowledge learned in school	3.43	3	3	0.937	−0.295
Articles in magazines about healthy lifestyle and healthy nutrition	3.20	3	3	1.082	−0.265
Product information, leaflets	2.83	3	3	1.027	0.057
Relevant pieces of writing in free publications	2.83	3	3	1.052	−0.027
Television programs	2.48	3	3	1.008	0.141
Television commercials	2.32	2	2	0.984	0.462
Advertisements in newspapers, on posters and billboards	2.31	2	2	0.940	0.358
Radio programs	2.30	2	2	0.935	0.355

**Table 4:** Component of electronic health literacy, component weights.

Statements	C_EHL_
I know how to use the Internet to answer questions about healthy nutrition.	0.756
I know where to find helpful resources on the Internet in my daily searches.	0.748
I can tell high-quality health resources of healthy nutrition from low-quality health resources on the Internet.	0.745
I know how to use the health information related to healthy nutrition found on the Internet to help me.	0.742
I know where to find helpful resources on the Internet in my daily searches.	0.731
I have the skills I need to evaluate the health resources of healthy nutrition I find on the Internet.	0.719
I feel confident in using the information about healthy nutrition from the Internet to make health decisions.	0.684
I know what health resources are available on the Internet related to healthy nutrition.	0.643

**Table 5:** Components of attitudes toward healthy nutrition and component weights.

Statements	C_AHN_	C_IHN_	C_RHNAA_
I pay close attention to healthy and balanced nutrition and choose my foods carefully.	0.843		
I make a conscious effort to find foodstuffs with ingredients that I consider beneficial.	0.760		
I pay attention to the effect of foods and foodstuffs on body weight.	0.742		
I make a conscious effort to avoid foods that are energy-dense (oil, sugar).	0.720		
I make a conscious effort to avoid foodstuffs with ingredients that I consider harmful.	0.688		
I try to create a balanced diet.	0.636		
When compiling my diet, I follow nutrition-related recommendations based on current medical knowledge.	0.623		
I persuade others, too, to eat a healthier diet.		0.879	
I find it important that the people around me should also have a healthier diet.		0.879	
I have tried several times to eat healthily but, after temporary self-punishment, I have returned to my usual routine.			0.693
Healthy eating always reminds me of slimming cures.			0.683
What tastes good makes me really healthy.			0.614
Knowledge about healthy nutrition keeps changing and expanding, thus it is difficult to keep up with it.			0.586
Healthy nutrition is very costly.			0.545

## Data Availability

The data presented in this study are available on request from the corresponding author. The data are not publicly available due to ethical restrictions.
